# Rewiring of glycerol metabolism in *Escherichia coli* for effective production of recombinant proteins

**DOI:** 10.1186/s13068-020-01848-z

**Published:** 2020-12-14

**Authors:** Chung-Jen Chiang, Yi-Jing Ho, Mu-Chen Hu, Yun-Peng Chao

**Affiliations:** 1 Department of Medical Laboratory Science and Biotechnology, China Medical University, No. 91, Hsueh-Shih Road, Taichung, 40402 Taiwan; 2 Department of Chemical Engineering, Feng Chia University, 100 Wenhwa Road, Taichung, 40724 Taiwan; 3 Department of Medical Research, China Medical University Hospital, Taichung, 40447 Taiwan; 4 Department of Food Nutrition and Health Biotechnology, Asia University, Taichung, 41354 Taiwan

**Keywords:** Metabolic engineering, Crude glycerol, Recombinant protein

## Abstract

**Background:**

The economic viability of a protein-production process relies highly on the production titer and the price of raw materials. Crude glycerol coming from the production of biodiesel is a renewable and cost-effective resource. However, glycerol is inefficiently utilized by *Escherichia coli*.

**Results:**

This issue was addressed by rewiring glycerol metabolism for redistribution of the metabolic flux. Key steps in central metabolism involving the glycerol dissimilation pathway, the pentose phosphate pathway, and the tricarboxylic acid cycle were pinpointed and manipulated to provide precursor metabolites and energy. As a result, the engineered *E*. *coli* strain displayed a 9- and 30-fold increase in utilization of crude glycerol and production of the target protein, respectively.

**Conclusions:**

The result indicates that the present method of metabolic engineering is useful and straightforward for efficient adjustment of the flux distribution in glycerol metabolism. The practical application of this methodology in biorefinery and the related field would be acknowledged.

## Background

The advent of recombinant DNA technology has revolutionized the biotechnology industry [1]. In particular, cells can be reprogrammed and instructed to express the protein of interest. This approach is acknowledged by its power for overproduction of pharmaceutical and industrial proteins in an efficient way. The annual market value of recombinant proteins reaches billions of dollars, and the global market still continues to grow [2]. Recent progress in identification of microbes with specific functions has accelerated the accumulation of genomic information [3–5], which facilitates exploration of more proteins applicable in industry and biorefinery. Accordingly, there arises a pressing need for an economically viable method to efficiently produce recombinant proteins.

*Escherichia coli* is recognized as the biotechnology workhorse because it has several advantages including easy manipulation with genetics, culturability with the cost-effective medium, and scalable production scheme [6]. Employment of *E. coli* that harbors the gene-born plasmid has been commonly applied for production of recombinant proteins based on glucose [7]. However, many problems associated with protein overproduction occur. For instance, the presence of a plasmid negatively affects cell proteins and ribosome components [8]. The gratuitous overexpression of β-galactosidase (β-Gal) was reported to cause the ribosome destruction in cells [9]. The folding process is usually afflicted with an abnormal level of the expressed protein, consequently forming inclusion bodies [10]. It is known that the physiological function of central metabolism provides precursor metabolites and energy to fuel the cellular activity. Most of cellular energy is utilized for the synthesis of proteins [11]. To force the production of a gene-encoding product perturbs cell physiology as a result of energy drainage and imbalanced carbon flux in central metabolism. This in turn triggers the global stress response which further restricts the cellular growth and protein production [12]. Moreover, the metabolic response of *E. coli* to protein overproduction is complicated and depends on the strain type as well as culture compositions [13]. Taken together, the task to produce a large amount of recombinant proteins remains challenging.

The production cost of a protein-production process is closely linked to the production titer and the price of raw materials. Crude glycerol exists in the waste stream from the production of biodiesel and is a renewable and cost-effective resource. Many studies have been focused on refinement of crude glycerol for the microbial production of value-added chemicals thus far [14]. Apparently, it would be biotechnologically sound to produce recombinant protein based on crude glycerol. The aerobic growth of *E. coli* on glycerol relies on metabolic pathways for dissimilation, gluconeogenesis, and glycolysis which are under the control of global regulators involving cAMP receptor protein (CRP), catabolite repressor/activator protein (Cra), and aerobic respiration control protein (ArcA) [15]. However, glycerol is less effectively utilized than glucose and liable to induce the carbon stress response in *E. coli* [16]. To address this issue, this study was aimed to effectively produce d-hydantoinase (HDT) by manipulation of key steps in metabolic pathways of glycerol to re-distribute the metabolic flux. Consequently, the engineered strain enabled efficient utilization of crude glycerol and greatly increased production of HDT. The result indicates the useful application of metabolic engineering to the field of recombinant protein production.

## Results

### Enhancement of the dissimilation pathway

This study was initiated with BAD-5 strain. It was previously constructed with the l-arabinose (l-Ara)-inducible T7 expression system [17]. Upon induction with l-Ara, this strain grown on glucose enabled production of the recombinant protein in an efficient and homogenous manner. It bears a genomic copy of T7 gene *1* (encoding T7 RNA polymerase) under the control of the *araBAD* promoter (P_*BAD*_). To ensure the persistent inducibility of l-Ara, l-Ara catabolism of the strain is nullified by removal of *araBAD* operon. The additional deletion of *ptsG and araFGH* in the strain aims to decouple the glucose-mediated catabolite repression of P_*BAD*_ and the l-Ara transport-induction loop, respectively. Finally, the enhanced expression of *araE* facilitates l-Ara uptake for the strain.

**Fig. 1:**
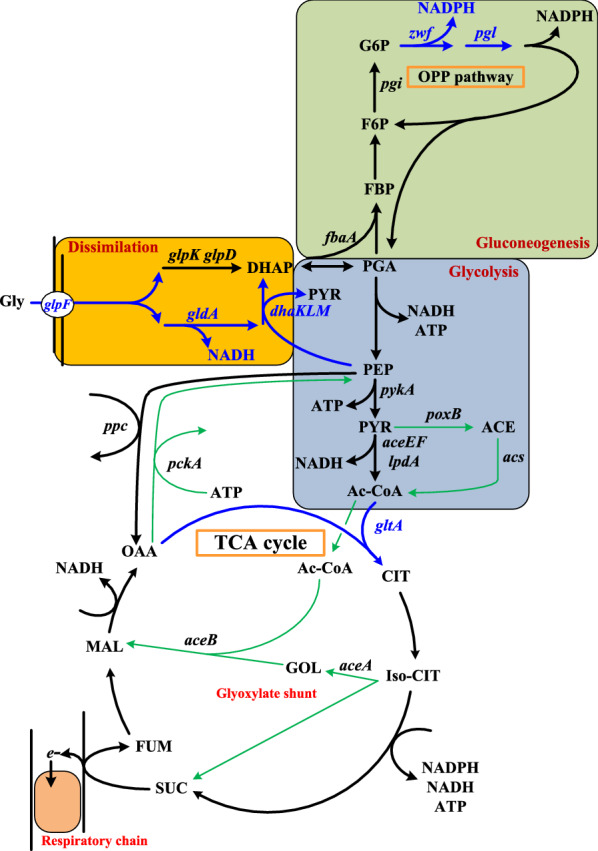
Central metabolic pathways of *E. coli* grown on glycerol. The metabolic pathways of enhancement and of the proposed acetate recycling were highlighted in blue and green, respectively. Genes involved in metabolic pathways include as follows: *aceA*, isocitrate lyase; *aceB*, malate synthase; *aceEF*-*lpd*, pyruvate dehydrogenase; *acs*, acetyl-CoA synthase; *citA*, citrate synthase; *dhaKLM,* dihydroxyacetone kinase*; fbaA*, fructose-biphosphate aldolase; *gldA*, glycerol dehydrogenase; *glpD*, glycerol 3-phosphate dehydrogenase; *glpK*, glycerol kinase; *glpF*, glycerol facilitator; *pckA*, PEP carboxykinase; *pgl*, lactonase; *pgi*, isomerse; *pykA*, pyruvate kinase; *poxB*, pyruvate oxidase; *ppc*, PEP carboxylase; *zwf*, glucose-6-phosphatase dehydrogenase. Abbreviations of metabolites: Ac-CoA, acetyl-CoA; ACE, acetate; CIT, citrate; DHAP, dihydroxyacetone phosphate; F6P, fructose-6-phosphate; FDP, fructose-diphosphate; FUM, fumarate; G6P, glucose-6-phosphate; Gly, glycerol; GOL, glyoxylate; Iso-CIT, isocitrate; MAL, malate; OAA, oxaloacetate; PEP, phosphoenolpyruvate; PGA, 3-phosphoglyceraldehyde; PYR, pyruvate; SUC, succinate

Figure 1 reveals that glycerol metabolism is generally fractionated into 3 module pathways: (1) the dissimilation pathway of glycerol, (2) the gluconeogenesis pathway of dihydroxyacetone phosphate (DHAP), and (3) the glycolysis pathway of DHAP. We attempted to manipulate the dissimilation pathway. The strain’s *glpF* was first fused with the *trc* promoter (P*trc*). Two catabolic pathways of glycerol exist in *E. coli*. The oxidative cleavage of glycerol proceeds via the *glpK*–*glpD* route while the *gldA*–*dhaKLM* route is utilized for the anaerobic fermentation of glycerol [18]. The anaerobic degradation pathway involves the phosphorylation reaction by phosphoenolpyruvate (PEP) and produces more NADH. The protein production is recognized to consume most of cellular energy. Therefore, the glycerol flux was directed to the *gldA*–*dhaKLM* pathway. Endogenous *gldA* and the *dhaKLM* operon in BAD-5 strain were fused with the λP_L_ promoter (PλP_L_) to activate the pathway activity without anaerobiosis. This genetic construction resulted in N31 strain which displayed an eightfold and threefold increase in the GldA (ca. 0.96 U/mg protein) and DhaKLM activity (ca. 0.015 U/mg protein), respectively.

**Fig. 2:**
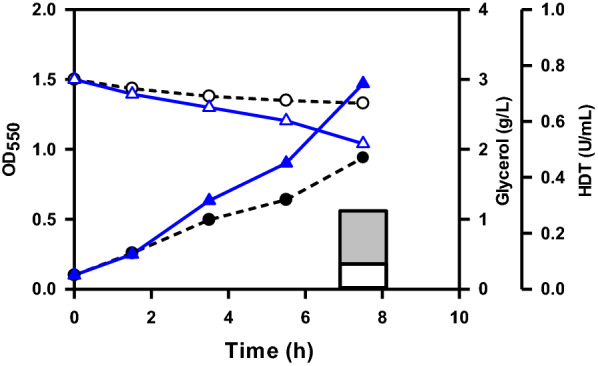
The performance of the strain engineered with glycerol metabolism. The experiment was carried out as described. Typical profiles were shown for N31/pET-TrChHDT strain with cell density (black triangle), glycerol consumption (white triangle), and the volumetric activity of HDT (solid bar) and for BAD-5/pET-TrChHDT strain with cell density (black circle), glycerol consumption (white circle), and the volumetric activity of HDT (empty bar)

HDT was chosen for illustration because it has no function interfering with cell physiology. This enzyme is of industrial importance for its usefulness to prepare d-*p*-hydroxyphenylglycine which is required for the synthesis of amoxicillin, a β-lactam antibiotic [19]. The performance of N31 strain was investigated for production of HDT by induction with l-Ara. Pure glycerol was used during the course of strain development. Plasmid pET-TrChHDT was transformed into the strain to obtain N31/pET-TrHDTCh strain which was cultured in a shake flask containing glycerol. BAD-5 strain bearing plasmid pET-TrChHDT (BAD-5/pET-TrChHDT) was employed as a control. As compared to BAD-5/pET-TrChHDT strain, N31/pET-TrChHDT strain consumed more glycerol and produced soluble HDT with a 1.2-fold increase in the activity (Fig. 2).

### Enhancement of the gluconeogenic pathway

**Fig. 3:**
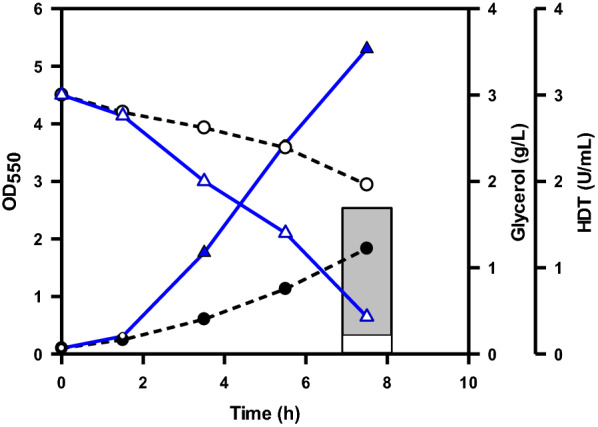
The performance of the strain engineered with gluconeogenesis metabolism. The experiment was carried out as described. Typical profiles were shown for N31-5(HDT) strain with cell density (black triangle), glycerol consumption (white triangle), and the volumetric activity of HDT (solid bar) and for N31(HDT) strain with cell density (black circle), glycerol consumption (white circle), and the volumetric activity of HDT (empty bar)

NADPH is required for biosyntheses. The gluconeogenic pathway of DHAP is linked to the oxidative pentose phosphate (OPP) pathway which provides NADPH (Fig. 1). It was previously illustrated to produce more reducing equivalents in the strain by redirection of carbon flux into the OPP pathway as a result of enhancing the expression level of *zwf* and *pgl* [20]. Glucose 6-phosphate (G6P) dehydrogenase encoded by *zwf* catalyzes the first step in the OPP pathway. N31 strain derived from *E. coli* B which lacks *pgl* (encoding lactonase) [21]. Therefore, N31 strain was engineered by fusion of PλP_L_ with *zwf* and recruitment of the PλP_L_-driven *pgl*. The construction resulted in N31-5 strain which showed a 2- and 4.1-fold increase in Zwf (ca. 8.2 U/mg protein) and Pgl activity (ca. 1.6 U/mg protein), respectively. To remain stable, N31 and N31-5 strains were equipped with a genomic copy of the P_T7_-driven HDT to give N31(HDT) and N31-5(HDT) strains, respectively. The resulting strains were cultured and induced for the HDT production. As shown in Fig. 3, N31-5(HDT) strain displayed better growth and consumed more glycerol than N31(HDT) strain. The activity of soluble HDT in N31-5(HDT) strain was increased by sixfold relative to that for N31(HDT) strain.

### Forced direction of the glycolytic flux

**Fig. 4:**
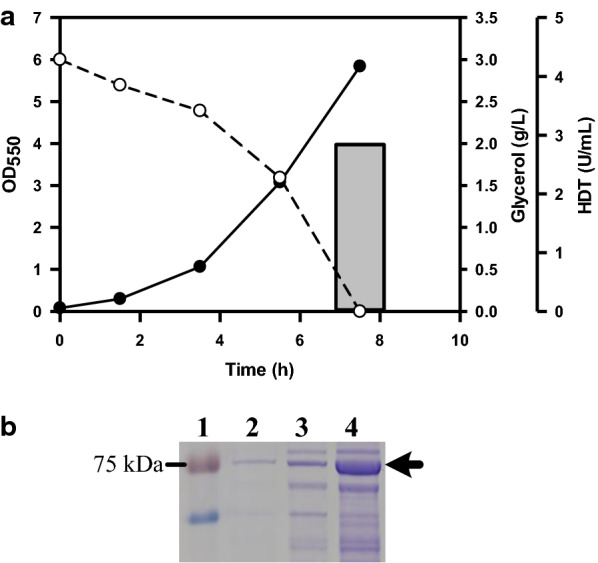
**a** The performance of the strain engineered with the TCA cycle. The experiment was carried out using crude glycerol. Typical profiles were shown for N31-5AK(HDT) strain with cell density (black circle), glycerol consumption (white circle), and the volumetric activity of HDT (solid bar). **b** The SDS-PAGE analysis of soluble proteins from recombinant strains in crude glycerol. At the end of the experiment, bacterial culture of 1 mL were harvested and processed for the analysis of SDS-PAGE as described. The arrow indicated the position of soluble HDT. Keys: lane 1, protein marker; lane 2, proteins of BAD-5/pET-TrChHDT strain; lane 3, proteins of N31(HDT) strain; lane 4, proteins of N31-5AK(HDT) strain

The flux from the glycolysis pathway of DHAP enters the tricarboxylic acid (TCA) cycle, an indispensible pathway which provides reducing equivalents and precursors for biosyntheses (Fig. 1). Its operation mode consists of the oxidative or reductive route in response to oxygen availability [22]. Citrate synthase (encoded by *gltA*) catalyzes the synthesis of citrate from acetyl-CoA and oxaloacetate (OAA). The TCA cycle is regulated through the control of GltA [23]. As previously illustrated, the production of reducing equivalents was reduced in the TCA cycle by lowering the GltA activity [24]. To increase the activity of the TCA cycle, the PλP_L_-driven *gltA* of *Corynebacterium glutamicum* CCRC 11384 (Cg*gltA*) was introduced into N31-5(HDT) strain. The construction gave N31-5AK(HDT) strain which gained a 2.1-fold increase in the GltA activity. The shake-flask culture of N31-5AK(HDT) strain was carried out for the protein production. The result showed that the strain performed equally well in either glycerol or crude glycerol (Table 1). As shown in Fig. 4a, the strain consumed all crude glycerol and enabled production of soluble HDT with the activity reaching 2.84 U/mL. Its HDT production reached 12% of total cell proteins (Fig. 4b). In comparison with BAD-5/pET-TrChHDT strain, N31-5AK(HDT) strain displayed an increase in the intracellular NADH/NAD^+^ (ca. 0.38) and NADPH/NADP^+^ ratios (ca. 1.64) by 25% and 30%, respectively.

**Table 1:** Summary of the production kinetics for strains with engineered traits

Strain	△*S*	OD (*T*_0.5_)	HDT	Y	Manipulated gene						
					*arcA*	*zwf*	*pgl*	*gldA*	*dhaKLM*	*gltA*	*glpF*
Pure glycerol											
BAD-5	0.04 ± 0.00	0.28 ± 0.01 (1.54)	0.09 ± 0.01	225	−	−	−	−	−	−	−
N31	0.13 ± 0.01	0.43 ± 0.02 (1.35)	0.19 ± 0.01	216	−	−	−	+	+	−	+
N31(HDT)	0.13 ± 0.01	0.53 ± 0.02 (1.32)	0.22 ± 0.01	244	−	−	−	+	+	−	+
N31-5(HDT)	0.32 ± 0.02	1.55 ± 0.05 (0.91)	1.68 ± 0.13	672	–	+	+	+	+	−	+
N31-Arc(HDT)	0.37 ± 0.02	1.61 ± 0.06 (0.91)	2.59 ± 0.19	948	△	+	+	+	+	−	+
N31-5AK(HDT)	0.40 ± 0.03	1.71 ± 0.08 (0.84)	2.83 ± 0.22	969	–	+	+	+	+	+	+
Crude glycerol											
N31-5AK(HDT)	0.40 ± 0.03	1.76 ± 0.09 (0.84)	2.84 ± 0.22	969							

Note that BAD-5 and N31 strains harbored plasmid pET-TrChHDT for expression of HDT. Glycerol consumption rate (∆*S*) in g/L h, final biomass (OD) in g dry cell weight (DCW)/L, the protein production (HDT) in U/mL, and the protein yield based on glycerol (Y) in U/g were calculated based on experimental data. Numbers in parentheses indicate the doubling time (*T*_0.5_) in h of strains

Symbols for manipulated genes: +, enhancement; −, wild type, △, deletion

## Discussion

Glucose is commonly employed for the production of recombinant proteins in *E. coli*. It has an advantage of easy dissimilation through central metabolism which provides precursor metabolites and energy to fuel cellular activities. Therefore, considerable efforts have been devoted to achieve the efficient production of target proteins based on glucose. Acetate overflow appears to be a frequently encountered event in the protein-overproducing *E. coli* which is incapable of properly coordinating glucose metabolism. This phenomenon is generally ascribed to the lower activity of the TCA cycle and respiratory chain relative to the uptake rate of glucose [25]. Metabolic engineering, thus, emerges as an enabling technology to address this issue. As successfully illustrated, acetate could be reduced in *E. coli* by over-expressing the anaplerotic enzyme (i.e., PEP carboxylase) to direct more carbon flux to OAA and deletion of *fadR* for activation of the glyoxylate shunt [26]. A similar approach was conducted with the expression of heterologous pyruvate carboxylase, and the β-Gal production improved by 68% [27]. Replacement of the phosphotransferase system-based glucose transport by galactose permease lowered glucose uptake and reduced acetate formation, consequently leading to fourfold higher production of green fluorescent protein [28]. In response to oxygen availability, the two-component system involving *arcA* and *arcB* mediates the anaerobic repression of enzymes in the TCA cycle, glyoxylate shunt, and others [29]. Inactivation of ArcA and/or ArcB reduced acetate yield and increased the β-Gal production by 30% [30]. Global regulators function to control metabolic pathways in connection with the physiological status of bacteria. Interestingly, mutant strains carrying a single knockout in the global regulator, including *arcA*, *arcB*, *cra*, *crp*, *cya*, *fnr*, or *mlc*, were shown to produce less acetate at the expense of glucose uptake [31].

Glycerol dissimilation is less efficient than glucose in *E. coli*. There are few efforts investigating the bacterial production of proteins based on glycerol. Overproduction of poorly folded proteins (i.e., inclusion bodies) is prone to induction of the stress response. To address this issue, the supplement of glycerol in the culture medium was proven to be useful for improving solubility of recombinant proteins [32, 33]. In addition, the application of glycerol as the glucose substitute for the recombinant protein production enabled reduction of acetate formation in *E. coli* [34]. As compared to *E. coli K*, *E. coli B* exhibits a higher activity in the TCA cycle and glyoxylate shunt due to the lower expression level of *arcA* and *iclR* [35]. This metabolic characteristic renders B strain less efficient in the production of acetate when grown on glucose. Alternatively, a study reported that an unusually high level of *poxB*, *acs, pckA*, and genes in glyoxylate shunt (*aceA* and *aceB*) was observed in *E. coli* during aerobic growth on glycerol [16]. These induced genes mediate the “carbon stress-based acetate-recycling mechanism” [16]. Acetate is synthesized by PoxB and then re-assimilated via the catabolic pathway consisting of *acs*, glyoxylate shunt genes, and *pckA*, consequently leading to an undetectable level of acetate.

In contrast to these previous studies, this work was conducted by rewiring glycerol metabolism of *E. coli B* strain to improve the protein production. Aerobic glycerol metabolism is limited by the GlpK activity [36]. Nevertheless, the *gldA*–*dhaKLM* route responsible for the oxygen-limited glycerol metabolism was targeted for improvement. As compared to its parent strain (BAD-5/pET-TrChHDT), N31/pET-TrChHDT strain exhibited a 2.3-fold increase in glycerol consumption. The increased glycerol flux was directed more to HDT (i.e., 100% more activity) than to biomass (i.e., 40% more) (Table 1). The result indicates that the engineered *gldA*–*dhaKLM* pathway is functional to augment the ability of the strain for aerobic utilization of glycerol, which produces more NADH (Fig. 1). Interestingly, the performance of N31 strain remained roughly unaffected when bearing either the multicopy plasmid (ColE1 origin) containing HDT (i.e., N31/pET-TrChHDT) or a genomic copy of HDT (i.e., N31(HDT)) (Table 1). Metabolic burden occurs in the cell overexpressing the plasmid-encoded gene [37, 38]. Therefore, the approach by integration of the target gene into the cell genome may alleviate the plasmid-caused adverse effect. The previous study reported that the level of *fbaA* and *pgi* increased in the strain grown on glycerol [16], which suggests that gluconeogenesis metabolism is necessary for provision of fructose 6-phosphate (F6P) and G6P from DHAP. The OPP pathway provides precursor metabolites and produces more reducing equivalents. Therefore, *zwf* and *pgl* were overexpressed to direct the flux through G6P into the OPP pathway. The resulting strain (N31-5(HDT)) metabolized 1.4-fold more glycerol than its parent strain (N31(HDT)). The increased consumption of glycerol was utilized to increase the synthesis of biomass and HDT by 2.1- and 6.6-fold (Table 1), respectively. Expression levels of genes in the OPP pathway remained unaffected in *E. coli* irrespective of glycerol or glucose [16, 39]. Nevertheless, the current result suggests that the OPP pathway presents to be a bottleneck in the aerobic metabolism of glycerol. Note that the engineered dissimilation of glycerol via the *gldA*–*dhaKLM* route diverts PEP to pyruvate. An elevated level of *pykA* encoding pyruvate kinase occurs in *E. coli* during growth on glycerol [16, 39]. Accordingly, PEP produced in the *glpK*–*glpD* route is in part converted to pyruvate by PykA. Pyruvate is further oxidized to acetyl-CoA by the reaction of pyruvate dehydrogenase and via the “acetate-recycling pathway”. *E. coli* grown on glycerol displays an unusually high level of *pckA* encoding PEP carboxykinase that converts OAA to PEP and serves to complete the cycling pathway for acetate reuse [16]. Therefore, available OAA and acetyl-CoA were directed into the TCA cycle by enhanced expression of citrate synthase in N31-5(HDT) strain. The resulting strain (N31-5AK(HDT)) enabled consumption of 25% more glycerol, contributing to 70% more HDT activity and 10% more biomass (Table 1). In a parallel study, the HDT production could also be improved in N31-5(HDT) strain deprived of *arcA* (i.e., N31-Arc(HDT)) but was inferior to that for N31-5AK(HDT) strain (Table 1). Derepression of genes in the TCA cycle by null *arcA* can improve the cycle activity [29]. Taken together, the current result suggests that GltA is a limiting step of the TCA cycle in the aerobic metabolism of glycerol. Finally, Table 1 revealed that enhancement of the OPP pathway greatly improved biomass, glycerol utilization, and the HDT production, while additional enhancement of the TCA cycle further improved the HDT production.

## Conclusion

In this work, key steps in the glycerol dissimilation pathway, the PP pathway, and the TCA cycle were identified and manipulated to rewire glycerol metabolism. This approach improves the production of biomass by 4.5-fold and of the HDT activity by 30-fold (N31-5AK(HDT) vs. BAD-5/pET-TrChHDT strain) (Table 1). The result clearly indicates that re-distribution of glycerol flux in central metabolism is very efficient for production of recombinant proteins (refer to Y in Table 1). In particular, specific genes instead of global regulators are targeted for engineering. This is in sharp contrast to the method of reprogramming global regulators as commonly employed for the production of recombinant proteins using glucose. Modification of global regulators usually leads to a pleiotropic effect on cell physiology, which is complex and unpredictable. In contrast, the present method of metabolic engineering is useful and straightforward for efficient adjustment of the flux distribution in glycerol metabolism. In summary, a modern biorefinery is composed of network and cascade processes aimed at production of value-added bioproducts (including enzymes, chemicals, and antibiotics) and biofuels [40]. This crude glycerol-based production platform for recombinant proteins would have a potential application in biorefinery.

## Methods

### Genetic manipulation

Plasmid pET-TrChHDT carried the fusion gene consisting of *trxA*, HDT gene, and the chitin-binding domain (ChBD) under the control of the T7 promoter (P_T7_), and was constructed as follows. The DNA containing HDT-ChBD was amplified from plasmid pChHDT [41] by PCR with primers (cgaattccatatggatatcatcatcaagaacg and gtgactggtgagtactcaacc). By the EcoRI–ScaI digestion, the PCR DNA was incorporated into plasmid pET-32a (Novagen Co.) to give plasmid pET32-TrChHDT. The fusion gene was recovered from plasmid pET32-TrChHDT treated with the ScaI–XbaI cut and then incorporated into plasmid pET20b (Novagen Co.) to obtain plasmid pET-TrChHDT. Note that TrxA improves the solubility of HDT and ChBD facilitates the immobilization of HDT [41].

The DNA containing the fusion gene linked to P_T7_ was recovered from plasmid pET-TrChHDT subjected to the NdeI–SacI digestion. The recovered DNA was incorporated into plasmid pPhi80-Km [42], resulting in plasmid pPhi-TrHDTCh. This plasmid was applied for insertion of the P_T7_-driven fusion gene into the strain’s genome following the reported protocol [42].

The DNA containing P*trc* was amplified from plasmid pTrc99A by PCR with Ta1–Ta2 primers (ctgggaattcagcttatcatcgactgcac and attccacacattatacgagc). By digestion with EcoRI, the PCR DNA was incorporated into the EcoRI and SmaI sites of plasmid pPL-Kan [24]. The construction gave plasmid pTrc-kan which carried the LE*-*kan*-RE*-P*trc* cassette. The passenger DNA contained LE*-*kan*-RE*-P*trc* with two homologous regions of *glpF* and was obtained from plasmid pTrc-kan by PCR with Ta3-Ta4 mega primers (acaacccggtaccgaggaattcagcaatgcactggcctttcaaggttgatgttt-gactcatatggatcaacctccttagg and tgcctacaagcatcgtggaggtccgtgactttcacgcatacaacaaaca-ttaactcttcaggatcccccattaatgtcgac). To enhance their expression level, *gldA* and *dhaKLM* genes were fused with PλP_L_. PCR was applied to amplify LE*-*kan*-RE*-PλP_L_ cassettes flanked with homologous extensions (passenger DNAs) from plasmid pPL-Kan with Gld1–Gld2 and Dha1–Dha2 primers according to the previous report [24]. Passenger DNAs were then electroporated into the strain and underwent homologous recombination by the act of λRed. Meanwhile, passenger DNAs were amplified from plasmid pPR-zwf and plasmid pSPL-pgl with primers RC11417/RC11418 and RC13034/RC13035, respectively [20]. By a similar approach, *zwf* and *pgl* were fused with PλP_L_. The integrated *kan* marker was later removed by Cre.

The PλP_L_-driven *gltA* of *C. glutamicum* CCRC 11384 was constructed in several steps. The gene was first amplified by PCR with primers (cacatatgtttgaaagggatatcgtg and tagggcccttagcgctcctcgcgagg). The PCR DNA was treated by *Apa*I–*Nde*I cut and then incorporated into plasmid pLoxTrc (Lab collection) to obtain plasmid pLoxTrc-GltA. Treated with the BamHI–NdeI cut, Cg*gltA* was recovered from plasmid pLoxTrc and then ligated into plasmid pLoxKm-PR (Lab collection) to give plasmid pPR-CgltA. Finally, the DNA containing the fusion of PλP_L_ with Cg*gltA* was amplified by PCR with primers (actatggatcccgcgaaattaatacgac and tagggcccttagcgctcctcgcgagg). The BamHI-treated PCR DNA was spliced into plasmid pLam-LoxKm [43] which was digested by BamHI and NruI to obtain plasmid pLam-PrCgltA. The PλP_L_-driven Cg*gltA* was integrated into the strain’s genome at λ attachment locus following the reported protocol [43].

The deletion of *arcA* was carried out by λRed-mediated homologous recombination of the PCR DNA (∆*arcA*:: FRT-*kan*-FRT) after electroporation. This DNA fragment was amplified from JW4364-1 strain [44] with the primers (gaaagtacccacgaccaagc and tgacccgtaatatcgactgg). The *kan* marker flanked by FRT was later removed using Flp.

### Bacterial culturing

The cell density was measured turbidimetrically at 550 nm (OD_550_). The recombinant strain was grown on LB medium (10-g/L Tryptone, 5-g/L yeast extract, and 5-g/L NaCl) with or without ampicillin (30 μg/mL) overnight. The overnight culture was seeded into Erlenmeyer flasks (125 mL) containing 15-mL M9Y medium (6-g/L Na_2_HPO_4_, 3-g/L KH_2_PO_4_, 0.5-g/L NaCl, 1-g/L NH_4_Cl, 1-mM MgSO_4_, 0.1-mM CaCl_2_, 2-g/L yeast extract) plus 3-g/L glycerol or crude glycerol (Great Green Technology Co., Taiwan). According to the manufacturer, the main composition of crude glycerol was 82% (w/w) glycerol, 1.7% fatty acids, and 4.6% ash. Ampicillin (15 μg/mL) was additionally supplemented in the medium for the strain that harbored the gene-born plasmid. The initial cell density was maintained at OD_550_ of 0.08. The bacterial culture was incubated at 37 °C in a rotary shaker set at 200 rpm. Upon reaching around 0.3 at OD_550_, bacteria were induced for the protein production by adding of l-Ara (30 μM) to the culture medium. The bacterial growth was monitored along the time course and the protein production was terminated after the induction was administrated at 6 h. Glycerol was analyzed using High-Performance Liquid Chromatography (HPLC) based on the previous report [45]. The intracellular NADH/NAD and NADPH/NADP ratios were measured with assay kits (ab65348 and ab65349, Abcam, UK.), and the procedure essentially followed the manufacturer’s instructions.

### Protein analysis

The bacterial culture (1 mL) was harvested by centrifugation, and cell pellets were resuspended in 0.1-M Tris-HCl buffer (0.2 mL) at pH 8.0. Cells were disrupted by sonication, followed by centrifugation. The supernatant was recovered and saved as the cell-free extract (CFX). The total protein content of CFX per culture volume (mg/mL) was estimated based on Bio-Rad Protein Assay reagent with bovine serum albumin (BSA) as a standard. CFX (20 μL) was applied for the protein analysis by sodium dodecyl sulfate-polyacrylamide gel electrophoresis (SDS-PAGE). Using Image Analyzer GAS9000 (UVItech), the resolution of SDS-PAGE was analyzed to estimate the amount of HDT. The HDT activity was assayed based on the previous report [46]. CFX (10 μL) was added to the reaction solution (1 mL) consisting of 0.1-M Tris-HCl buffer (pH 8.0), 6-mM d,l-hydroxyphenyl hydantoin (HPH), and 0.5-mM MnCl_2_. The reaction proceeded at 40 °C for 15 min and was terminated by heating the solution at 100 °C for 10 min. The concentration of un-reacted HPH was determined by HPLC and used to calculate the activity of HDT based on the protein (U/mg protein) in the reaction solution, where U was defined as μmol/min. The total activity per culture volume (i.e., volumetric activity (U/mL)) of HDT was obtained by multiplying the activity (U/mg) with the total protein content (mg/mL).

### Enzyme assay

Activities of endogenous proteins were determined essentially following the previous report [24]. In brief, harvested cells were resuspended in 1-mL saline solution and CFX was prepared in a similar way. The measurement of enzyme activities was conducted by adding CFX (100 µL) to each reaction solution (1 mL) at 30 °C. The GldA and DhaKLM activities were determined by monitoring the reduction of NAD^+^ from glycerol at 340 nm. The activity of G6P dehydrogenase (encoded by *zwf*) and of lactonase (encoded by *pgl*) were determined by monitoring the reduction of NADP^+^ from G6P at 340 nm. Moreover, the activity of citrate synthase (encoded by *gltA*) was measured by conversion of acetyl-CoA and OAA to citrate.

## Data Availability

Not applicable.

## Abbreviations

l-Ara: l-arabinose
CFX: Cell-free extract
ChBD: Chitin-binding domain
DHAP: Dihydroxyacetone phosphate
F6P: Fructose 6-phosphate
G6P: Glucose 6-phosphate
β-Gal: β-galactosidase
HDT: d-hydantoinase
HPLC: High-performance liquid chromatography
ΗPH: d,l-hydroxyphenyl hydantoin
OPP: Oxidative pentose phosphate
OAA: Oxaloacetate
PEP: Phosphoenolpyruvate
SDS-PAGE: Sodium dodecyl sulfate polyacrylamide gel electrophoresis
TCA: Tricarboxylic acid
